# The intrinsic macrolide resistome of *Escherichia coli*

**DOI:** 10.1128/aac.00452-24

**Published:** 2024-06-28

**Authors:** Yibing Ma, Mattia Pirolo, Bimal Jana, Viktor Hundtofte Mebus, Luca Guardabassi

**Affiliations:** 1 Department of Veterinary and Animal Sciences, University of Copenhagen, Frederiksberg, Denmark; 2 Center for Genomic Medicine, Massachusetts General Hospital, Boston, Massachusetts, USA; Shionogi Inc., Florham Park, New Jersey, USA

**Keywords:** *E. coli*, intrinsic resistance, tilmicosin, macrolide, TraDIS

## Abstract

Intrinsic resistance to macrolides in Gram-negative bacteria is primarily attributed to the low permeability of the outer membrane, though the underlying genetic and molecular mechanisms remain to be fully elucidated. Here, we used transposon directed insertion-site sequencing (TraDIS) to identify chromosomal non-essential genes involved in *Escherichia coli* intrinsic resistance to a macrolide antibiotic, tilmicosin. We constructed two highly saturated transposon mutant libraries of >290,000 and >390,000 unique Tn5 insertions in a clinical enterotoxigenic strain (ETEC5621) and in a laboratory strain (K-12 MG1655), respectively. TraDIS analysis identified genes required for growth of ETEC5621 and MG1655 under 1/8 MIC (*n* = 15 and 16, respectively) and 1/4 MIC (*n* = 38 and 32, respectively) of tilmicosin. For both strains, 23 genes related to lipopolysaccharide biosynthesis, outer membrane assembly, the Tol-Pal system, efflux pump, and peptidoglycan metabolism were enriched in the presence of the antibiotic. Individual deletion of genes (*n* = 10) in the wild-type strains led to a 64- to 2-fold reduction in MICs of tilmicosin, erythromycin, and azithromycin, validating the results of the TraDIS analysis. Notably, deletion of *surA* or *waaG*, which impairs the outer membrane, led to the most significant decreases in MICs of all three macrolides in ETEC5621. Our findings contribute to a genome-wide understanding of intrinsic macrolide resistance in *E. coli*, shedding new light on the potential role of the peptidoglycan layer. They also provide an *in vitro* proof of concept that *E. coli* can be sensitized to macrolides by targeting proteins maintaining the outer membrane such as SurA and WaaG.

## INTRODUCTION

Macrolides inhibit bacterial protein synthesis by binding to the 23S rRNA of the 50S subunit of ribosome (1). They are primarily effective against Gram-positive bacteria, whereas most Gram-negative bacteria are intrinsically resistant due to the low permeability of bacterial outer membrane, which limits antibiotic entry into the cell (1, 2). Mutations at the L4 and L22 ribosomal proteins or 23S rRNA can also give rise to non-transferable resistance to macrolides (1, 3). Additionally, macrolide resistance may involve the expression of acquired genes encoding target site-modifying rRNA methylases (*erm*), drug-inactivating phosphorylases (*mph*), or efflux pumps (*mef* and *msr*) (3, 4). Acquisition of transferrable resistances is a known phenomenon that increases erythromycin and azithromycin MICs in human and veterinary clinical *Escherichia coli* to concentrations above 1,024 mg/L (4–6). However, the genes and molecular networks mediating intrinsic resistance, as well as the biological imperative driving the acquisition of transferrable macrolide resistance genes, have not been elucidated.

Transposon directed insertion-site sequencing (TraDIS) is a popular method that combines transposon mutagenesis with next-generation sequencing to study gene-phenotype connections at the genome scale (7, 8). It has been used to evaluate essential genome (8, 9) and to identify non-essential genes that are involved in specific biological traits such as antimicrobial resistance or susceptibility (10–12). To the best of our knowledge, however, no studies have yet applied TraDIS or similar genome-wide approaches to investigate macrolide-gene interaction in clinical *E. coli*. In the present study, we constructed highly saturated transposon mutant libraries in *E. coli* K12 MG1655 and, for the first time, in a clinical enterotoxigenic *E. coli* (ETEC) strain. We performed TraDIS to evaluate the contribution of individual non-essential genes to intrinsic resistance to tilmicosin (TIL), a 16-membered macrolide, leading to identification of genes whose inactivation significantly reduced bacterial growth in the presence of TIL. As proof of concept, we demonstrated that deletion of these genes sensitizes *E. coli* to different subclasses of macrolides, including TIL, erythromycin (ERY) (14-membered), and azithromycin (AZI) (15-membered).

## RESULTS

### Whole-genome sequencing reveals multiple resistance genes in ETEC strain ETEC5621

Whole-genome sequencing (WGS) of ETEC5621 revealed a 5.03-Mb chromosome exhibiting an average GC content of 50.66%. Annotation of the genome identified 4,868 genes including 92 tRNA and 22 rRNA genes. Among these, 4,034 genes were found to be shared with the laboratory *E. coli* K12 strain MG1655, accounting for 85% and 88% of their respective genomes. Six extrachromosomal plasmids were identified in ETEC5621 with sizes of 6, 46, 61, 61, 88, and 114 kb, respectively. Genome analysis identified two of the plasmids harboring multiple resistance genes conferring resistance to macrolides [*mph*(A)], aminoglycosides [*aph* (*6*)*-Id*, *aph*(*3′*)*-Ia*, and *aadA24*], β-lactams (*blaTEM-30*), and lincomycin [*lnu*(G)].

### Construction of saturated Tn5 mutant library for ETEC5621 and MG1655

Tn5 mutant libraries were constructed for both *E. coli* ETEC5621 and MG1655 strains. Each library was estimated to contain >250,000 individual mutants. Sequencing of libraries with biological replicates generated >12 million and >14 million reads for ETEC5621 and MG1655, respectively. Mapping reads to the genomes led to identification of >290,000 and >390,000 unique transposon insertions with an average of 1 transposon insertion per 19 and 12 nucleotides in the genome of ETEC5621 and MG1655, respectively. The full list of parameters of TraDIS data set for all sequencing libraries (*n* = 14) is provided in Table S1. As shown in Fig. S1, the Tn5 insertion profile depicting the distribution of insertions across the genomes of both strains confirmed the saturation of the libraries. Gene essentiality analysis identified 540 (11% of the genome) and 347 (8% of the genome) essential genes in ETEC5621 and MG1655, respectively, among which 235 genes were shared within the two strains.

### TraDIS identifies genes required for growth in the presence of TIL

**Fig 1:**
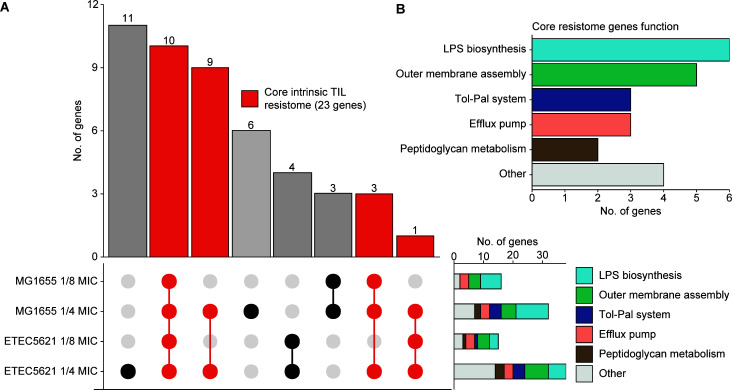
Overview of genes required for growth of *E. coli* MG1655 and ETEC5621 after exposure to tilmicosin (TIL) at different concentrations. (**A**) UpSet plot showing intersections between strains and treatment combinations is displayed in a matrix layout, and each column corresponds to a specific section in a traditional Venn diagram. The histogram shows the number of unique and shared genes at each circle or intersection, respectively. Connected circles in the matrix indicate shared genes, and unconnected circles represent unique genes. Core genes of the intrinsic macrolide resistome are illustrated in red. (**B**) Main functions of the genes in the core resistome.

TraDIS analysis identified non-essential genes conferring intrinsic resistance to TIL. ETEC5621 and MG1655 exhibited a TIL MIC of 128 and 64 µg/mL, respectively. The contribution of non-essential genes to TIL resistance was evaluated by measuring depletion of transposon insertions following exposure to TIL at 1/4 MIC and 1/8 MIC, which caused partial growth inhibition of libraries (Fig. S2). Genes with a fourfold or more decrease in the transposon insertions [log_2_ fold change (logFC) ≤−2 and false discovery rate (FDR)-adjusted *P* value (*q* value) ≤0.05] after TIL exposure compared to the unexposed control were considered important for growth with TIL. As shown in Fig. 1A, TraDIS analysis identified 38 genes important for growth of ETEC5621 with TIL at 1/4 MIC, of which 15 genes were also required for growing at 1/8 MIC. A similar pattern was observed for MG1655, as all the genes identified at 1/8 MIC (*n* = 16) were also identified at 1/4-MIC condition (*n* = 32). The combined resistome of both strains resulted in a comprehensive set of 47 genes, showcasing an enrichment in functions including lipopolysaccharide (LPS) biosynthesis (*n* = 11), outer membrane assembly (*n* = 8), Tol-Pal system (*n* = 5), efflux pump (*n* = 3), and peptidoglycan metabolism (*n* = 3) (Fig. 1A). The full list of these genes is provided in the Supplemental Data Set. TraDIS analysis also identified a significant depletion of transposon insertions (logFC = −3.45 and *q* value = 0) in the acquired macrolide resistance gene *mph*(A) in ETEC5621 after growing with 1/4 TIL MIC compared to the no-antibiotic control, but no difference was observed in the 1/8 TIL MIC.

A total of 23 genes were identified in both ETEC5621 and MG1655 and were collectively defined as the core macrolide resistome of *E. coli* (Fig. 1A; Table 1). The core resistome consisted of genes involved in LPS biosynthesis (*waaG*, *waaQ*, *gmhB*, *galU*, *waaP*, and *lapC*) (13–16), outer membrane assembly (*lptC*, *surA*, *bamB*, *lpp*, and *bepA*) (17–21), efflux pump (*acrA*, *acrB*, and *tolC*) (22), Tol-Pal system (*tolA*, *tolB*, and *tolR*) (23), peptidoglycan metabolism (*prc* and *nlpI*) (24, 25), and other functions (*pgm*, *hrpA*, *atpB*, and *rplI*) (Fig. 1B). Ten of these genes were also required for growth at the presence of 1/8-MIC TIL for ETEC5621 and MG1655 (Fig. 1A; Table 1). Fifteen and nine genes were identified only in ETEC5621 or MG1655 (Fig. 1A and Supplemental Data Set), respectively. These strain-specific genes were also functionally enriched in the biogenesis and maintenance of the cell envelope, including five genes in ETEC5621 and six genes in MG1655.

**TABLE 1:** Inactivated genes in mutants that were significantly depleted after exposure of *Escherichia coli* ETEC5621 and MG1655 to 1/4 MIC or 1/8 MIC of the macrolide tilmicosin^*a*^

Gene	Function	ETEC5621				MG1655			
		1/4 MIC		1/8 MIC		1/4 MIC		1/8 MIC	
		logFC	*q* value	logFC	*q* value	logFC	*q* value	logFC	*q* value
***lptC***	Lipopolysaccharide transport system protein LptC	−7.59	2.39E-95	−4.37	3.77E-71	−5.66	8.94E-30	−3.12	2.67E-12
***surA***	Chaperone SurA	−7.01	1.37E-07	−6.94	1.02E-06	−3.13	4.65E-08	−2.91	1.83E-07
*tolR*	Tol-Pal system protein TolR	−6.63	9.61E-06			−2.03	8.08E-05		
***acrA***	Multidrug efflux pump membrane fusion lipoprotein AcrA	−6.17	0	−3.70	0	−5.45	4.85E-98	−4.15	8E-62
***bamB***	Outer membrane protein assembly factor BamB	−6.00	3.87E-73	−4.13	7.39E-55	−6.75	1.73E-31	−2.42	1.21E-08
***acrB***	Multidrug efflux pump RND permease AcrB	−5.83	0	−3.36	0	−5.37	1.3E-149	−4.09	1.77E-83
***tolC***	Outer membrane channel of efflux systems	−5.53	1.41E-21	−4.19	4.89E-17	−4.41	1.47E-48	−4.59	1.53E-46
*lpp*	Murein lipoprotein	−5.52	2.28E-42			−7.26	2.36E-05		
*tolB*	Tol-Pal system periplasmic protein TolB	−4.86	5.82E-14			−2.27	4.29E-07		
*prc*	Tail-specific protease	−4.50	9.16E-14			−4.76	6.36E-18		
*nlpI*	Lipoprotein NlpI	−4.41	2.06E-17			−2.97	1.43E-10		
***waaG***	Lipopolysaccharide glucosyltransferase I	−4.31	1.24E-15	−4.18	1.12E-13	−2.04	0.002374	−2.06	3.62E-05
***lapC***	LPS homeostasis protein	−4.21	3.43E-14	−2.60	3.41E-77	−6.33	3.35E-40	−5.16	1.37E-33
*waaQ*	Lipopolysaccharide core heptosyltransferase 3	−3.69	0			−2.67	1.09E-48		
*gmhB*	D-glycero-beta-D-manno-heptose-1	−3.67	0.01596			−7.48	1.54E-06		
*galU*	UTP-glucose-1-phosphate uridylyltransferase	−3.56	0.03728			−2.50	0.011102		
*tolA*	Tol-Pal system protein TolA	−3.47	6.67E-18			−2.44	4.71E-07		
*rplI*	50S ribosomal subunit protein L9	−3.40	0.00138			−3.51	2.31E-11		
*hrpA*	ATP-dependent RNA helicase HrpA	−3.26	4.51E-21			−2.19	1.07E-17		
***pgm***	Phosphoglucomutase	−3.23	1.47E-16	−3.67	3.84E-17	−2.96	1.32E-08	−2.47	1.64E-06
***waaP***	Lipopolysaccharide core heptose (I) kinase	−3.09	3.07E-39	−2.17	6.98E-26	−5.31	9.35E-22	−3.89	1.11E-15
*bepA*	Beta-barrel assembly-enhancing protease	−2.48	2.59E-71			−4.97	7.98E-51		
*atpB*	ATP synthase F_0_ complex subunit a	−2.18	5.08E-10			−3.21	0.002464		

^*a*^ Genes identified at 1/8 MIC are highlighted in bold.

### Validation of selected genes identified by TraDIS

Twelve core resistance genes, comprising all 10 genes identified under 1/8-MIC condition and 2 additional genes associated with peptidoglycan metabolism (i.e., *prc* and *nlpI*), were selected to construct gene knockouts by lambda red recombineering (Table 1), and mutants of 10 genes were generated for both strains (Table 2). Deletion of *lapC* and *lptC* was unsuccessful possibly due to the essential region at the 5′ end of their coding sequences, where no transposon insertion was present (Fig. S3). In addition to TIL, we also included two additional macrolides used in human medicine, namely, ERY and AZI, in MIC testing for validation. In ETEC5621, deletion of *surA* or *waaG* resulted in the most significant reductions in MICs of TIL (from 128 to 16 µg/mL), ERY (from 1,024 to 128 µg/mL), and AZI (from 64 to 8 µg/mL). In MG1655, the highest MIC reductions were observed following deletion of *tolC* for TIL (from 64 to 1 µg/mL) and ERY (from 64 to 2 µg/mL), and deletion of *waaG* or *waaP* for AZI (from 4.0 to 0.125 µg/mL). Notably, no MIC reduction for any macrolide was observed upon deletion of *pgm* in MG1655 (Table 2). MIC determination for the *surA* mutant of MG1655 was unfeasible due to the mutant’s inability to exhibit visible growth in Mueller-Hinton broth (MHB) media. Once tested in Luria-Bertani (LB) media, Δ*surA* showed fourfold, twofold, and twofold reductions in MICs compared to the wild-type (WT) strain for TIL, ERY, and AZI, respectively. Five additional classes of antibiotics were tested in the WT strain and mutants of ETEC5621 to investigate drug-specific effects (Table S2). Deletion of *surA* resulted in a two- to eightfold reduction in MICs of ampicillin/sulbactam, tetracycline, ciprofloxacin, and colistin. Individual deletion of the *acrAB-tolC* genes led to a fourfold reduction in MICs of tetracycline and ciprofloxacin. No reduction in gentamycin MICs was observed in any of the mutants.

**TABLE 2:** MICs (µg/mL) of TIL, ERY, and AZI in the wild-type and gene-deletion mutants of ETEC5621 and MG1655^*b*^

Genotype	ETEC5621			MG1655		
	TIL	ERY	AZI	TIL	ERY	AZI
Wild type	128	1024	64	64	64	4
Δ*surA*	16	128	8	ND^*a*^	ND^*a*^	ND^*a*^
Δ*waaG*	16	128	8	8	8	0.125
Δ*waaP*	32	256	8	8	8	0.125
Δ*bamB*	32	256	32	8	8	1
Δ*pgm*	32	128	16	64	64	4
Δ*acrA*	64	512	64	4	2	0.25
Δ*acrB*	64	512	64	4	2	0.25
Δ*tolC*	64	512	64	1	2	0.25
Δ*prc*	32	512	32	16	16	1
Δ*nlpI*	32	512	16	32	16	2

^*a*^ ND, not determined in Mueller-Hinton broth. MIC values of wild-type MG1655 in LB correspond to 64, 64, and 16 µg/mL for TIL, ERY, and AZI, respectively. MIC values of MG1655 Δ*surA* mutant in LB correspond to 16, 32, and 8 µg/mL for TIL, ERY and AZI, respectively.

^*b*^ AZI, azithromycin; ERY, erythromycin; TIL, tilmicosin.

**Fig 2:**
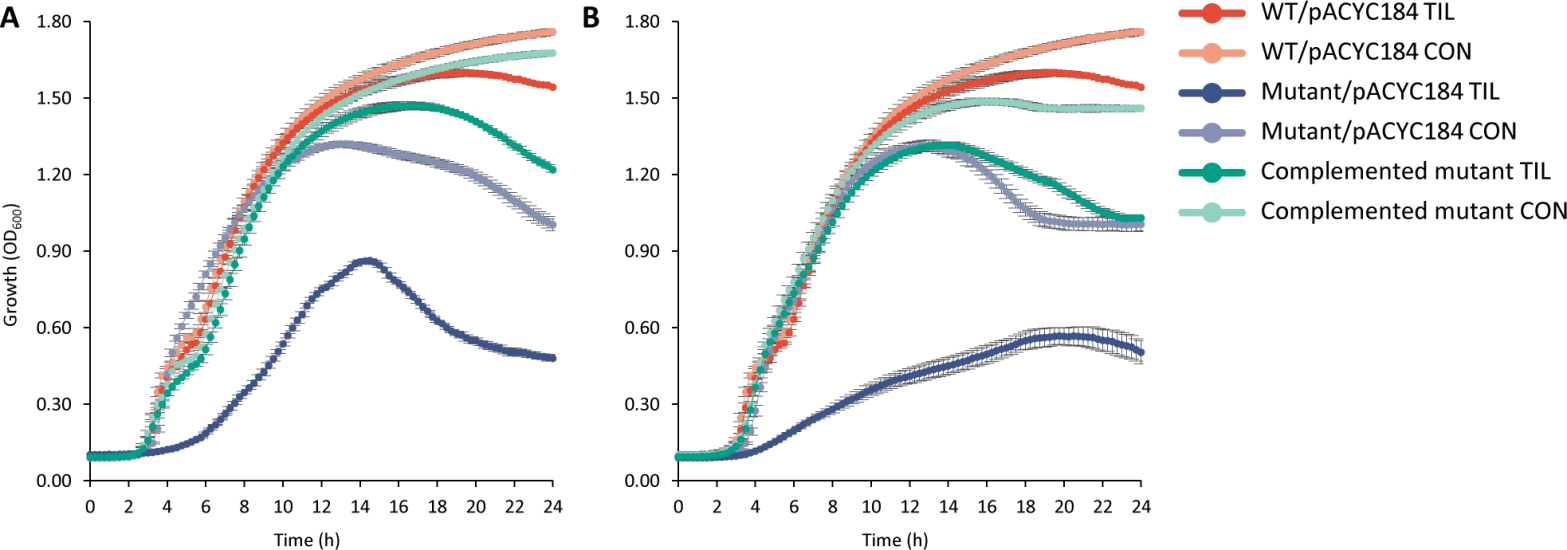
Growth curves of the wild-type (WT), mutant, and complementation strains of *surA* (**A**) and *waaG* (**B**) in ETEC5621. Optical density at 600 nm (OD_600_) was measured over a 24-h growth period in Luria-Bertani (LB) broth control (CON) and LB broth supplemented with 8-µg/mL of tilmicosin (TIL). Data are the mean ± standard deviation of triplicate experiments.

**Fig 3:**
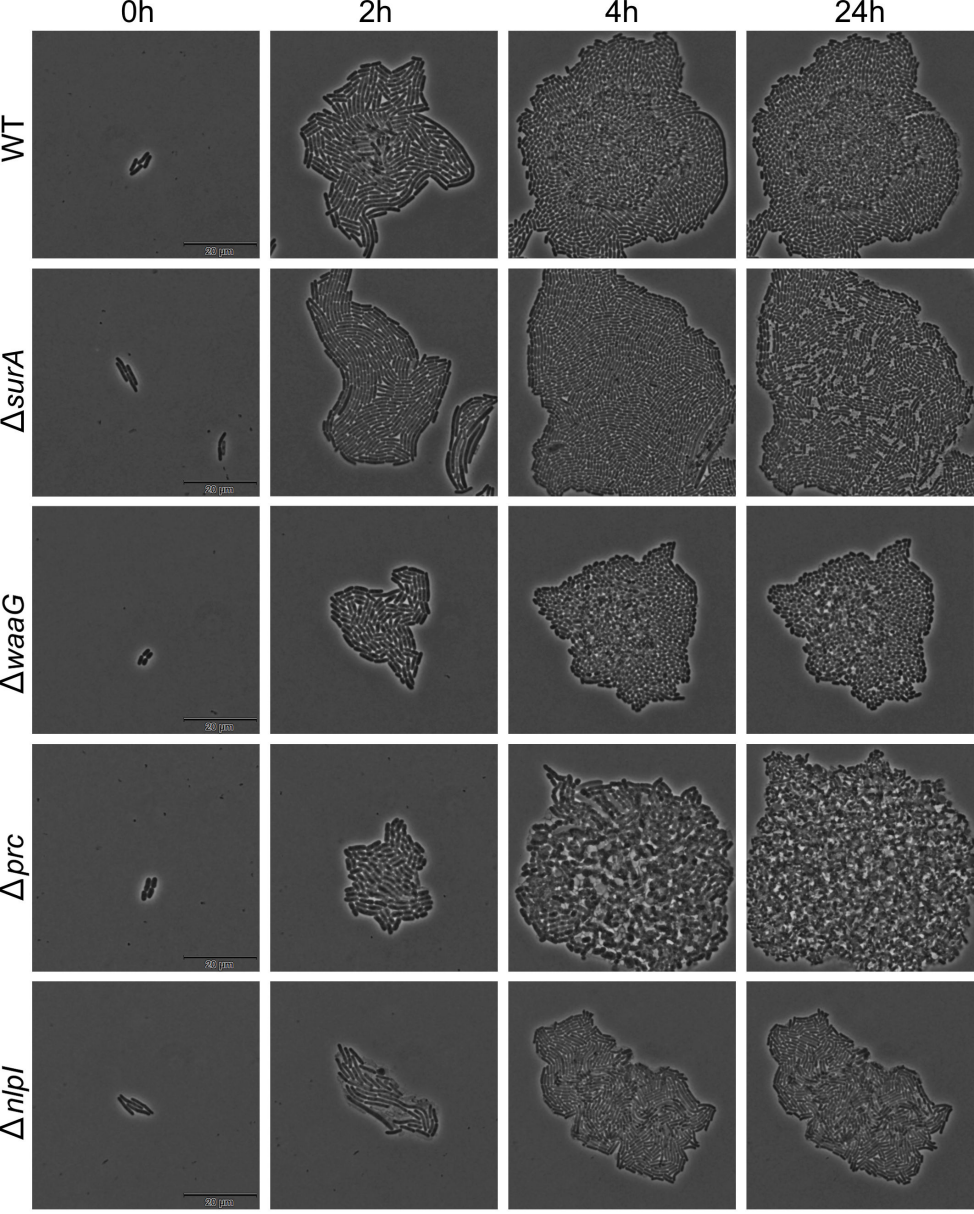
Simultaneous time-lapse microscopy of ETEC5621 wild-type (WT) strain and mutant derivatives Δ*surA*, Δ*waaG*, Δ*prc*, and Δ*nlpI*, grown on Luria-Bertani media supplemented with 1.2% agarose at 37°C. Three positions were imaged per strain, and phase-contrast images were acquired every 5 minutes for 24 h. Here 0, 2, 4 and 24 h are depicted for each strain.

The two mutants showing the highest MIC reduction for all three tested macrolides in the clinical strain, Δ*surA* and Δ*waaG*, were selected for further investigation, revealing 128-, 32-, and 16-fold reductions of the MBC compared to the WT strain for TIL (from 2,048 to 16 µg/mL), ERY (from 4,096 to 128 µg/mL), and AZI (from 128 to 8 µg/mL), respectively. Notably, MBC values overlapped with MIC values of these mutants, denoting a bactericidal effect of macrolides (bacteriostatic in *E. coli*). Upon complementation in both Δ*surA* and Δ*waaG* mutants by pACYC184, the MICs and MBCs were fully or partially restored to the values observed in the WT strain (Table 3). We also investigated the effect of *surA* and *waaG* deletions on the growth of ETEC5621 in the LB medium with or without 8-µg/mL TIL, which corresponded to 1/2-MIC for Δ*surA* and Δ*waaG*. As shown in Fig. 2, Δ*surA* and Δ*waaG* showed a slight growth defect compared to the WT strain at the stationary phase in the absence of TIL. In the presence of 8-µg/mL of TIL, however, deletion of *surA* or *waaG* impaired the growth. Δ*surA* appeared slower to come out of the exponential phase and then reached the stationary phase at 30% of cell density compared to the WT strain, while Δ*waaG* had a slower growth rate than the WT. The growth defects observed in the mutants were effectively restored by complementation of the respective genes. To further elaborate on the effect on growth caused by disruption of different cell envelope components, we performed simultaneous time-lapse microscopy in Δ*prc* and Δ*nlpI* (peptidoglycan), in addition to Δ*surA* (outer membrane proteins) and Δ*waaG* (LPS). Interestingly, inactivation of either *surA*, *waaG*, *prc*, or *nlpI* resulted in distinct observable growth phenotypes, including increased stationary phase autolysis (Δ*prc* and Δ*surA*), increased spherical cell morphology (Δ*waaG*), early exponential phase autolysis (Δ*nlpI*), and cell elongation (Δ*surA* and Δ*nlpI*) (Fig. 3).

**TABLE 3:** MICs (µg/mL) and MBCs (µg/mL) of TIL, ERY, and AZI in the wild-type, mutant, and complementation strains of *surA* and *waaG* in ETEC5621^*a*^

Strain	TIL		ERY		AZI	
	MIC	MBC	MIC	MBC	MIC	MBC
WT	128	2,048	1,024	4,096	64	128
WT/pACYC184	128	2,048	1,024	4,096	64	128
Δ*surA*/pACYC184	16	16	128	128	8	8
Δ*surA*/pACYC184::*surA*	128	128	512	512	64	64
Δ*waaG*/pACYC184	16	16	128	128	8	8
Δ*waaG*/pACYC184::*waaG*	128	2,048	512	1,024	64	128

^*a*^ AZI, azithromycin; ERY, erythromycin; TIL, tilmicosin.

### Testing of known SurA and WaaG inhibitors

We searched for commercially available inhibitors of SurA and WaaG in *E. coli*, leading to the identification of Fmoc-L-tryptophan and Fmoc-L-phenylalanine for SurA (26), and 4-(2-amino-1,3-thiazol-4-yl)phenol for WaaG (27). To explore the potential to use these compounds as helper molecules to potentiate TIL, we performed checkboard assays to assess their interactions with TIL in ETEC5621 and MG1655. Growth of both strains was inhibited by 4-(2-amino-1,3-thiazol-4-yl)phenol with a MIC of 4 mM, but no synergy with TIL was observed across the tested concentrations (0–8 mM) via checkerboard assays. In the tested range of concentration (0–4 mM), Fmoc-L-tryptophan and Fmoc-L-phenylalanine had no antimicrobial activity or synergistic interaction with TIL.

## DISCUSSION

Intrinsic resistance to macrolides in *E. coli* is traditionally associated with the natural barrier function of the outer membrane. However, the understanding of the molecular mechanisms responsible for this resistance phenotype remains elusive. To expand the knowledge of the underlying mechanisms, we conducted a genome-wide mapping of gene-macrolide interactions in two *E. coli* genetic backgrounds, including a clinical enterotoxigenic strain. The results revealed the existence of a core resistome composed by 23 genes implicated in intrinsic macrolide resistance. Among these genes, we identified *acrAB* and *tolC*, supporting the notion that the AcrAB-TolC efflux system is responsible for intrinsic resistance to various antimicrobials, including macrolides (22, 28). Eleven genes (nearly half of the core resistome) are directly related to LPS biosynthesis (*n* = 6) or outer membrane assembly (*n* = 5), confirming the essential role of the outer membrane in intrinsic macrolide resistance. Genes encoding for the inner membrane and periplasmic components of the Tol-Pal system, namely, *tolA*, *tolB*, and *tolR*, were also involved in the intrinsic macrolide resistome. This system plays a pivotal role in maintaining outer membrane and cell wall homeostasis, and deletion of its components is known to result in pleiotropic phenotypes with increased antimicrobial sensitivity (23, 29). Of particular interest, we also identified two genes (i.e., *prc* and *nlpI*) that encode a proteolytic system responsible for regulation of peptidoglycan metabolism (25), indicating a new insight into the involvement of the peptidoglycan layer in intrinsic macrolide resistance. Peptidoglycan biogenesis is a dynamic machinery requiring synthases for cross-linked glycan strand formation and hydrolases for cross-link cleavage to enable expansion (25, 30). In *E.coli*, peptidoglycan hydrolysis is stringently regulated by a pathway where the periplasmic protease Prc coupling its adaptor protein NlpI degrades the main peptidoglycan hydrolase MepS (25, 31). Inactivation of the Prc/NlpI pathway resulted in hyper-accumulation of MepS (25) and other peptidoglycan-cleaving enzymes negatively regulated by this proteolytic system, such as MepH (24), MltB, and DigH (32), leading to continuous peptidoglycan cross-link hydrolysis and severe cell wall defect. As peptidoglycan maturation also modulates the biosynthesis and assembly of outer membrane proteins (33), we deduce that Prc/NlpI inactivation compromises cell envelope integrity, thereby increasing macrolide permeation into the cytoplasm. This notion was further supported by the severe impact on cell morphology and onset of cellular autolysis observed after inactivation of *prc* or *nlpI* (Fig. 3). Finally, four genes in the core resistome were assigned to other functions that are not directly related to the cell envelope or efflux pump. Identification of these genes aligns with previous observations indicating that their deletion in *E. coli* (*hrpA* and *rplI*), *Salmonella* Typhimurium (*pgm*), or *Staphylococcus aureus* (*atpB*) results in increased antimicrobial susceptibility (34–37). Our results add to the understanding of the role played by these genes in macrolide resistance phenotype, which was not investigated in the previous studies.

The number of intrinsic resistance genes increased following exposure to higher macrolide concentrations, indicating that *E. coli* employs a complex mechanism involving various biological processes and cellular components (e.g., the Tol-Pal system and peptidoglycan layer) in response to high selective pressure. Despite their intrinsic resistance, these commensal bacteria inhabiting the intestinal tract of humans and warm-blooded animals face the challenge of being exposed to high macrolide concentrations due to the poor oral bioavailability of these antibiotics, e.g., 25% for erythromycin and 37% for azithromycin (38). This biological imperative may drive the evolution of *E. coli* to develop mechanisms to withstand the effects of macrolides, including the acquisition of transferrable macrolide resistance genes. In essence, the complex response of *E. coli* to macrolides is likely to reflect its evolutionary adaptation to survive and thrive in environments where these antibiotics are present at high concentrations, highlighting the intricate interplay between bacterial biology, environmental factors, and selective pressure imposed by antimicrobial use.

We observed decreased macrolide MICs in single-gene deletion mutants in 10 core genes involved in the intrinsic macrolide resistome. Interestingly, deletion of *acrAB-tolC* resulted in the most significant MIC reductions (16- to 64-fold) in the laboratory strain in contrast with the minimal MIC reductions (0- to 2-fold) observed in the clinical strain. Cytoplasmic concentration of antibiotics is determined by the balance between the rate of permeation through the cell envelope (influx) and the rate of extrusion by the efflux system (efflux) (39, 40). Presumably, the two *E. coli* strains used in the present study display different scenarios in this balance for macrolides, where influx contributes more significantly to the intrinsic resistance in the clinical strain, while efflux plays a dominant role in the laboratory strain. In addition, MICs in the clinical strain were generally higher than that in the laboratory strain, likely due to the presence of the acquired macrolide resistance gene *mph*(A) in the clinical strain. While our previous study suggested that *mph*(A) contributes to ERY and AZI resistance but not to TIL resistance (4), TraDIS analysis identified a significant depletion of transposon insertions in *mph*(A) compared to the control after exposure to 1/4 MIC of TIL but not 1/8 MIC, indicating that the *mph*(A)-encoded phosphotransferase inactivates TIL, albeit with limited efficiency. This might also account for the mere twofold difference in TIL MIC observed between MG1655 (64 µg/mL) and ETEC5621 (128 µg/mL), despite both MICs falling within the *E. coli* WT population.

We also observed the strain-specific phenotype of gene deletion mutants derived from the two strains. Deletion of *pgm* led to MIC reductions in the clinical strain ETEC5621 but not in the laboratory strain MG1655. The effect of *pgm* deletion on antimicrobial susceptibility in *S*. Typhimurium was ascribed to reduced O-antigen production (36), which is not applicable to *E. coli* K-12 strains as they are unable to synthesize O-antigen on their lipopolysaccharide (41). This is in line with a previous study showing that a *pgm* mutant of another *E. coli* K12 strain (BW25113) displayed identical erythromycin susceptibility compared to the WT (42).

Our results show that SurA and WaaG are promising drug targets for sensitizing clinical *E. coli* to macrolides. SurA is a periplasmic chaperone required for biosynthesis and proper assembly of outer membrane proteins, while WaaG is a glycosyltransferase involved in the LPS core synthesis (13, 43). The significant reduction of MIC levels observed upon the inactivation of *surA* or *waaG* makes these two gene products putative targets for developing macrolide helper drugs. In principle, a compound that inhibits the activity of SurA or WaaG could enhance macrolide efficacy by not only increasing *E. coli* sensitivity to macrolides at low concentrations but also rendering macrolides bactericidal against *E. coli*, leading to rapid elimination of bacteria. Helper drugs interfering with SurA and WaaG would have a broad spectrum as these proteins are highly conserved in *E. coli*, *Salmonella*, and other Gram-negative bacteria, with sequence identities above 85% (43, 44). Moreover, they could serve as potentiators of diverse antimicrobial classes since *surA* mutants in *Pseudomonas aeruginosa* and *waaG* mutants in *Salmonella enterica* have been shown increased susceptibility to β-lactams and fluoroquinolones (45, 46).

Despite these promising results, none of the commercially available inhibitors tested in this study displayed synergy with TIL. Bell et al. identified three inhibitors of the *E. coli* SurA, namely, Fmoc-β-(2-quinolyl)-D-alanine, Fmoc-L-tryptophan, and Fmoc-L-phenylalanine, based on *in silico* prediction and *in vitro* affinity assays (26). Muheim et al. demonstrated that 4-(2-amino-1,3-thiazol-4-yl)phenol bound to *E. coli* WaaG and inhibited its activity *in vitro* (27). However, no data on the efficacy of these compounds in *E. coli* have been provided. When testing the interactions between TIL with Fmoc-L-tryptophan, Fmoc-L-phenylalanine, and 4-(2-amino-1,3-thiazol-4-yl)phenol in ETEC5621 and MG1655, we observed no synergy. In addition, Fmoc-L-tryptophan and Fmoc-L-phenylalanine displayed insolubility in water and low solubility in DMSO, while 4-(2-amino-1,3-thiazol-4-yl)phenol was poorly soluble in water. The solubility issue of these compounds not only limited the concentration range tested in our study but also represented a challenge for their use to sensitize *E. coli* to TIL. We speculated that the periplasmic (for SurA) or cytoplasmic (for WaaG) concentration of active compounds was difficult to achieve to the level required for micromolar binding affinity as shown *in vitro*. Nevertheless, these compounds could be regarded as scaffolds for developing lead molecules demonstrating enhanced solubility and synergy with TIL and other macrolides.

The results of our study carry significant clinical implications regarding the potentiation of azithromycin’s efficacy against Gram-negative enteric pathogens (e.g., ETEC, *Salmonella* spp., and *Shigella* spp.) since this macrolide is recommended as one of the first choices for treatment of traveler’s diarrhea (47). There has been no available susceptibility breakpoints of macrolides for *E. coli*. Deletion of *surA* or *waaG* decreased azithromycin MIC values from 64 to 8 μg/mL, which is below the azithromycin breakpoints for *Salmonella* and *Shigella* (≤16 µg/mL) (48, 49). Additionally, our results are relevant for exploring innovative strategies to manage ETEC infections in pig veterinary practice. ETEC is the most common cause of post-weaning diarrhea, a widespread disease that significantly impacts morbidity and mortality in pig production (50). Effective management of post-weaning diarrhea has become challenging due to the recent restrictions in the use of zinc oxide and colistin, which were traditionally used for treatment of this disease (51, 52). Moreover, resistance to the current first-line antibiotic, neomycin, is rising due to the increased use of this aminoglycoside and the spread of neomycin resistance plasmids across ETEC lineages (53, 54). Sensitization of *E. coli* to macrolides could be a rapid and cost-effective strategy to address this emerging challenge in veterinary medicine. TIL is already marketed as feed premix of pigs for the treatment and prevention of pneumonia caused by *Pasteurella multocida* and *Actinobacillus pleuropneumoniae* and enteritis caused by *Lawsonia intracellularis* (42, 55). In a previous study, we demonstrated that certain peptidomimetics sensitize porcine clinical *E. coli* isolates to TIL at concentrations that could be achieved in the pig intestinal tract after oral administration (4). In the present study, deletion of *surA* or *waaG* decreased TIL MIC values to 16 µg/mL, which is below the lowest TIL MIC value that we reported in porcine clinical *E. coli* isolates (32 µg/mL) (4), as well as below the TIL susceptibility breakpoints for Gram-positive pathogens such as *P. multocida* and *A. pleuropneumoniae* (≤ 16 µg/mL) (42). Taken together, these data indicate that repurposing of TIL could be a viable option to address the existing lack of effective antimicrobials for managing ETEC infections in pig farming.

We acknowledge the limitations of TraDIS, which may overlook essential regions in non-essential genes such as *lptC* and *lapC*, making the validation of results through gene deletion unfeasible. Given that these two genes were previously identified as essential in *E. coli* (56), we hypothesize that they are partially essential, and the non-essential part of these genes plays a crucial role in maintaining fitness during macrolide stress, which warrants further investigations. Furthermore, TraDIS cannot assess essential genes or intergenic regions involved in intrinsic macrolide resistance, and the genes identified in our study may not encompass the entire macrolide resistome of *E. coli* since only two strains were analyzed.

In conclusion, this study highlights that intrinsic macrolide resistance in *E. coli* depends on the functional integrity of the entire cell envelope, involving not only the outer membrane but also the peptidoglycan layer. The study also provides a proof of concept that intrinsic resistance could be reversed by inactivation of specific proteins, such as SurA and WaaG, leading to increased outer membrane permeability. This result supports the possibility of developing inhibitors targeting these proteins that can be used to repurpose macrolides against *E. coli*, thereby addressing the shortage of effective therapeutic options against this important human and veterinary pathogen.

## MATERIALS AND METHODS

### Strains, growth conditions, and reagents

Two strains were selected for TraDIS, the laboratory *E. coli* K-12 strain MG1655 and the clinical ETEC strain ETEC5621, which was isolated in 2019 from the gut of a pig suffering from post-weaning diarrhea in a Danish pig farm (57). The latter strain was selected to be representative of the most predominant ETEC lineage circulating in Danish herds (sequence type ST100 and fimbriae type F4) (54).

Strains were routinely grown at 37°C in LB broth (Becton Dickinson, Albertslund, Denmark) or on Luria agar (LA) plates (Becton Dickinson). MHB (Oxoid, UK) was used for antimicrobial susceptibility testing. Super optimal broth with catabolite repression (SOC) medium (Sigma-Aldrich, USA) was used for recovery of competent cells after transformation. Antibiotics including tilmicosin, erythromycin, azithromycin, trimethoprim, kanamycin, gentamicin, chloramphenicol, tetracycline, ciprofloxacin, and colistin sulfate salt were purchased from Sigma-Aldrich. EZ-Tn5 <DHFR-1> Insertion Kit and EZ-Tn <KAN-2>Tnp Transposome Kit were purchased from LGC Biosearch Technologies. Fmoc-L-tryptophan and Fmoc-L-phenylalanine were purchased from Tokyo Chemical Industry (Tokyo, Japan), and 100-mM solution were prepared from powder dissolved in dimethyl sulfoxide (DMSO) (Sigma-Aldrich). 4-(2-Amino-1,3-thiazol-4-yl)phenol was purchased from Sigma-Aldrich, and 1-M solution was prepared from powder dissolved in DMSO. GenElute Bacterial Genomic DNA Kit (Sigma-Aldrich) was used for genomic DNA extraction. Primers were synthesized in TAG Copenhagen and are listed in Table S3.

### Antimicrobial susceptibility testing

MIC was tested in MHB medium by broth microdilution following the Clinical and Laboratory Standards Institute (CLSI) protocol (58). MBC was tested following the CLSI protocol (59). MICs and MBCs were tested more than once, and consistent values from at least two biological replicates were presented.

### Growth curve assay

A single colony from fresh LA plate was inoculated into LB medium for overnight growth at 37°C. Overnight culture was diluted by sterile saline to McFarland 0.5, and further 1:100 was diluted into 200-µL fresh LB medium supplemented with or without antibiotic in wells of a 96-well microtiter plate. Each experiment was performed in three replicates. The prepared plate was covered with lid and placed in a microplate reader (BioTek). Absorbance at 600 nm [optical density at 600 nm (OD_600_)] in each well was measured every 15 minutes in a 24-h duration by setting a kinetic measurement with continuous orbital shaking at 37°C.

### Checkerboard assay

Interactions between TIL and compounds were tested by checkerboard assay performed as previously described (60) with some modifications. Briefly, TIL was twofold serially diluted along the rows in a 96-well microtiter plate, while the compound was twofold serially diluted along the columns to create a matrix in which each well contained a combination of both agents at different concentrations. The interaction of the two compounds was interpreted as synergy, antagonism, or indifference for fractional inhibitory concentration index values of ≤0.5, >4.0, and >0.5–4.0, respectively.

### WGS and analysis

The genome of ETEC5621 was sequenced by a combination of short- and long-read sequencing. For short-read sequencing, genomic DNA was extracted from overnight culture and then quantified and qualified by using a NanoDrop 1000 (Thermo Fisher Scientific, USA) and a Qubit v.2.0 fluorometer (Thermo Fisher Scientific). DNA libraries were constructed by using the Nextera XT library preparation kit (Illumina, USA) following the manufacturer’s protocol, with subsequent sequencing on the MiSeq platform (Illumina).

For long-read sequencing, high-molecular-weight DNA extraction was performed using the Monarch HMW DNA Extraction Kit for Tissue (New England Biolabs, Massachusetts, USA) following the manufacturer’s instructions. Nanopore library preparation was performed using the Rapid Barcoding Kit 96, SQK-RBK110-96 (Oxford Nanopore Technologies, Oxford, UK). Sequencing was performed on an Mk1C MinION platform on a Flow Cell R9.4.1 (Oxford Nanopore Technologies).

Raw reads from Illumina sequencing and Nanopore sequencing were assembled using Unicycler v.0.5.0 (61). Plasmids were identified by using PlasmidFinder v.2.1 (https://cge.food.dtu.dk/services/PlasmidFinder/) and were further confirmed by BLASTn analysis against the non-redundant nucleotide National Center for Biotechnology Information database. Antibiotic resistance genes were identified by using ResFinder v.4.3.3 (http://genepi.food.dtu.dk/resfinder). Chromosomal sequence was annotated by using PROKKA v.1.14.6 (62).

### Construction of TraDIS libraries

Competent cells were prepared as previously described (63) with slight modifications. In brief, overnight cultures were 1:100 diluted in LB medium. To enhance the electrotransformation efficiency of ETEC1655, polymyxin B nonapeptide (Sigma-Aldrich) in a final concentration of 2 µg/mL was additionally supplemented to the LB medium (64). Cells were grown to an OD_600_ of 0.5–0.6, then harvested and washed once with ice-cold water and twice with 10% glycerol, and finally resuspended in 10% cold glycerol.

EZ-Tn5 <DHFR-1>Tnp Transposome was prepared by using the EZ-Tn5 <DHFR-1> Insertion Kit following the manufacturer’s instructions. Then, 1-µL EZ-Tn5 <DHFR-1>Tnp Transposome or EZ-Tn5 <KAN-2>Tnp Transposome was electroporated into an aliquot of competent cells of ETEC5621 or MG1655, respectively. After electroporation, cells were recovered in 1-mL SOC medium for 1.5 h at 37°C and subsequently selected on LA plates supplemented with 20-µg/mL trimethoprim for ETEC5621 or 50-µg/mL kanamycin for MG1655. Colonies were collected into MHB supplemented with 20% glycerol and then stored at −80°C.

To check the saturation of the constructed libraries, genomic DNAs were extracted in duplicate from a library aliquot containing ~3 × 10^9^ Tn-mutant cells and then processed for TraDIS sequencing and analysis.

### Tilmicosin exposure and TraDIS sequencing

The MICs of TIL for ETEC5621 and MG1655 were determined before TraDIS. In duplicate, approximately 1 × 10^8^ Tn-mutant cells from each library were inoculated into 10-mL MHB medium supplemented with TIL in concentrations of 0, 1/8 MIC, and 1/4 MIC, respectively. Mutants were grown at 37°C for 24 h and then 1 mL of bacterial culture was used for genomic DNA extraction.

Two micrograms of extracted DNA in 100-µL H_2_O was sheared to an average fragment size of 300–400 bp using a sonication device (Bioruptor Pico, Diagenode) with the following profile: 10 cycles of 30-s on and 90-s off at low frequency. The subsequent steps of DNA library preparation was performed following the protocol described in the TraDIS Toolkit method (65), with TraDIS adapter and primers previously designed (65, 66). The resulting DNA was sequenced on an Illumina MiSeq platform using a MiSeq reagent kit v.2 (50 cycles) (Illumina), as previously described (65).

### TraDIS data analysis and statistics

TraDIS sequence reads were firstly processed by using a custom *fq2bam.pl* script to take the transposon tag from each read and add it to the front of the read, and then convert the file into SAM format (66). The produced SAM file was subsequently converted into BAM format by using Samtools v.1.19.2 (66). The next steps were performed following the Bio::TraDIS pipeline (https://github.com/sanger-pathogens/Bio-Tradis) (65). In brief, processed reads were mapped to the reference genome of ETEC5621 or MG1655 (GenBank accession number U00096.3) to determine read counts and unique transposon insertion sites per gene. Reads in the 10% of the 3′ end of each gene were trimmed out, as many essential genes appear to tolerate insertions toward the end of the coding sequence (65). Statistical analysis was carried out using R v.3.2.3 included in the Bio::Tradis pipeline. Gene essentiality was evaluated using the *tradis_essentiality.R* script. The logFC of read counts and *q* value for each gene between antimicrobial-exposed and unexposed samples were evaluated by using the *tradis_comparisons.R* script. We set cut-offs as logFC of ≤−2 and *q* value of ≤0.05 to select candidate genes important for growth of the strain under antibiotic exposure.

### Construction of gene deletion mutants

Genes of interest were individually deleted in ETEC5621 and MG1655 by employing the lambda red recombineering as previously described (67), with minor modifications. A Chl^R^ cassette flanked by upstream and downstream homologies to the adjacent chromosomal sequences of the target gene was PCR amplified from pKD3 plasmid, and then purified by gel extraction. The temperature-sensitive lambda red recombinase plasmid pKD46-Gm (68) was transformed into ETEC5621 and MG1655 via electroporation. Strains carrying the pKD46-Gm plasmid were incubated in LB medium supplemented with 20-µg/mL gentamicin and shaken at 28°C overnight, and then 1:100 diluted into fresh LB medium supplemented with 20-µg/mL gentamicin and 2-µg/mL polymyxin B nonapeptide. L-arabinose (Sigma-Aldrich) at a final concentration of 80 mM was added to the subculture when the OD_600_ reached 0.4. After 1.5 h of induction, the cells were made electrocompetent, and then the Chl^R^ cassette was introduced by electroporation. Recombined mutants were selected on LA plates containing 20-µg/mL chloramphenicol at 37°C and then verified by colony PCR using primers listed in Table S3.

### Gene complementation

A low-copy number plasmid, pACYC184, was used for gene complementation to mutants of interest. Briefly, the backbone of pACYC184 and the coding sequence of the target gene were PCR amplified using the primers listed in Table S3. After DNA purification, the plasmid backbone and gene insert were assembled by using the NEBuilder HiFi DNA Assembly Master Mix (New England Biolabs) according to the manufacturer’s protocol. The resulting complementary plasmid was transformed into the mutant, and transformants were selected on LA plates containing 10-µg/mL tetracycline and verified by colony PCR. The original pACYC184 plasmids were transformed into the WT strain and the mutants as controls.

### Simultaneous time-lapse microscopy

The *E. coli* strains of ETEC5621 WT, Δ*surA*, Δ*prc*, Δ*waaG*, and Δ*nlpI*, were grown in LB media for 3 h at 37°C before before being adjusted to OD_600_ ~0.05. A 2.8 × 1.7 cm Gene Frame (Thermo Scientific) was attached to a clean microscope slide, whereby a five-well construction was made within the Gene Frame using appropriately cut fragments of another Gene Frame. LB medium was supplied with 1.2% agarose and melted at 84°C for 30 minutes in a heating block. One-milliliter melted LB-agarose mixture was added to the Gene Frame and immediately leveled by applying a microscope slide on top of the melted agarose mix, then light pressure was applied. The microscope slide was carefully removed as the agarose mix solidified (2–3 minutes at room temperature). A microliter of OD_600_ ~0.05 adjusted *E. coli* strains was added to each individual well and let dry slightly under a flame. A 2.4 × 3.2 cm coverslip was applied on top and sealed by running the bottom of the scalpel along the edges. The microscope slide was inserted into an Olympus IX83 inverted motorized microscope. The temperature of the microscope was set to 37°C within a cellSens incubator module. Using the cellSens software, a program saving three positions per strain was created, whereby two autofocus steps were applied before phase-contrast imaging. Images were acquired every 5 minutes for 24 h, using a 100 × 1.4 N.A. oil-immersion objective and a sCMOS Photometrics Prime camera. Images were analyzed in ImageJ, and composite image was created from identical time points of 0, 2, 4, and 24 h.

## Data Availability

Whole-genome sequence of strain ETEC5621 and TraDIS sequencing reads have been submitted to the NCBI Sequence Read Archive (SRA) under BioProject accession no. PRJNA1090041.

## SUPPLEMENTAL MATERIAL

Data Set S1 aac.00452-24-s0001.xlsx Definition and function classifications of the entire set of intrinsic tilmicosin resistance genes.

Supplemental figures and tables aac.00452-24-s0002.docx Fig. S1 to S3; Tables S1 to S3.

ASM does not own the copyrights to Supplemental Material that may be linked to, or accessed through, an article. The authors have granted ASM a non-exclusive, world-wide license to publish the Supplemental Material files. Please contact the corresponding author directly for reuse.
