# Supplementary material for: The intrinsic macrolide resistome of Escherichia coli
Source: Antimicrob Agents Chemother. 2024 Jun 28;68(8):e00452-24. doi: 10.1128/aac.00452-24 (PMC11304742; doi:10.1128/aac.00452-24)
Supplement: Supplemental figures and tables — Fig. S1 to S3; Tables S1 to S3. [file aac.00452-24-s0002.docx]

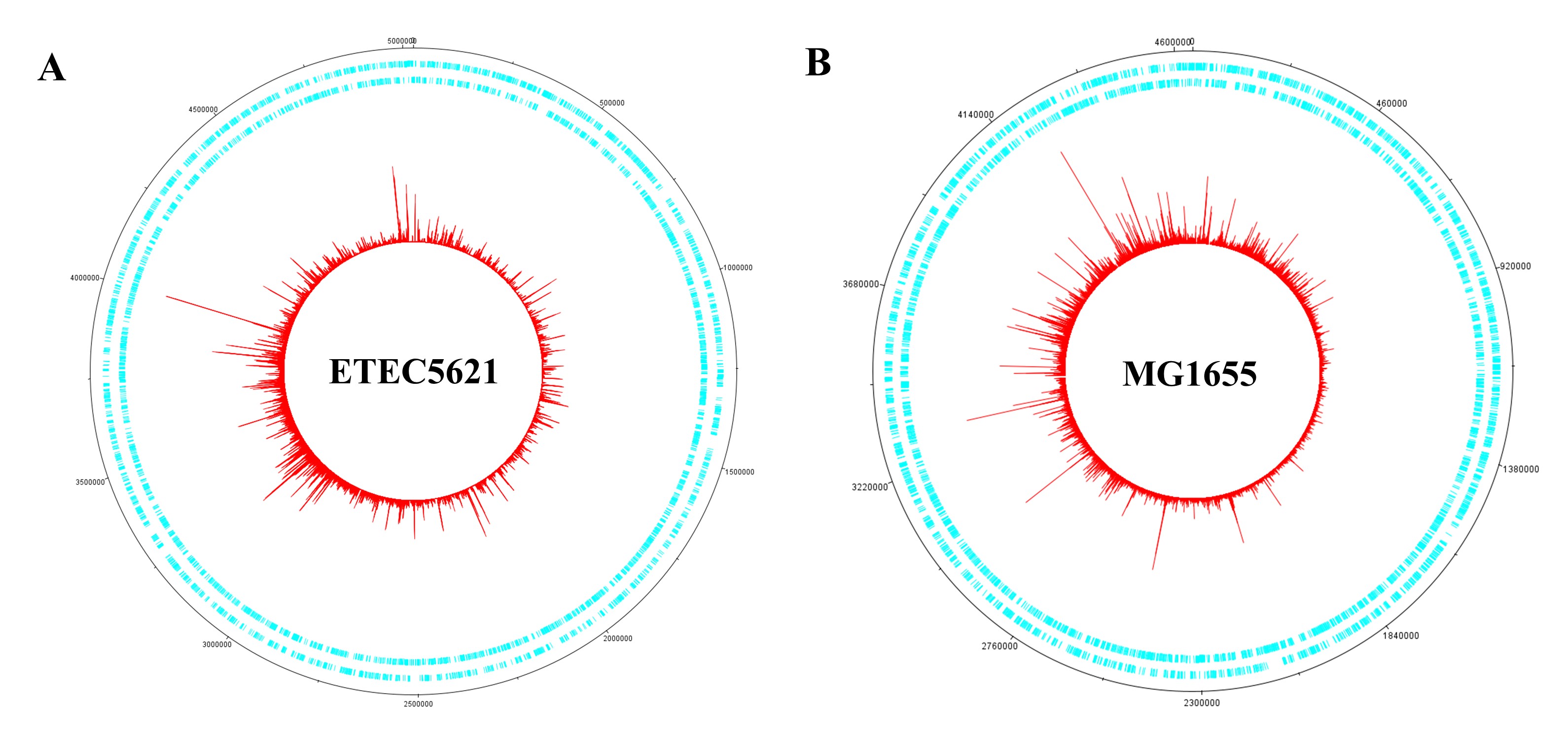


**FIGURE S1** Genome-wide transposon insertion sites mapped to the genome of E. coli ETEC5621 (A) and MG1655 (B). The black outermost track represents the E. coli genome, with base pairs marked from the annotation origin. The next inner track in cyan corresponds to the sense and antisense coding DNA sequences (CDS). The innermost circle displays red lines, indicating the frequency and locations of transposon insertions mapped onto the genome. The figure was generated using DNAPlotter (Artemis).


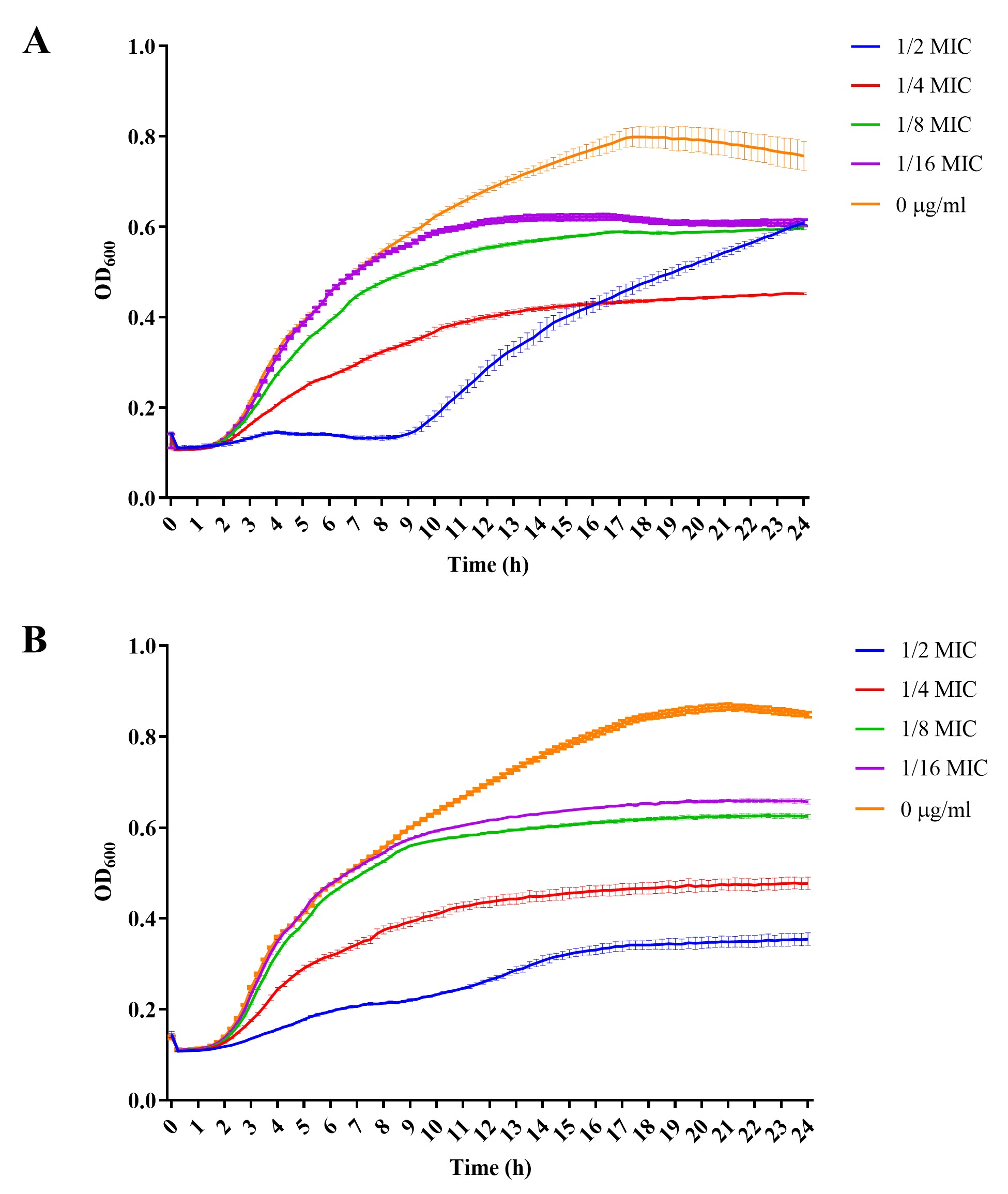


**FIGURE S2** Growth kinetics of transposon mutant library of ETEC5621 (A) and MG1655 (B). OD600 values were measured in MHB with or without TIL at different concentrations in 24 h. Three biological replicates were included for each sample and data are the mean ± standard deviation of the triplicates.


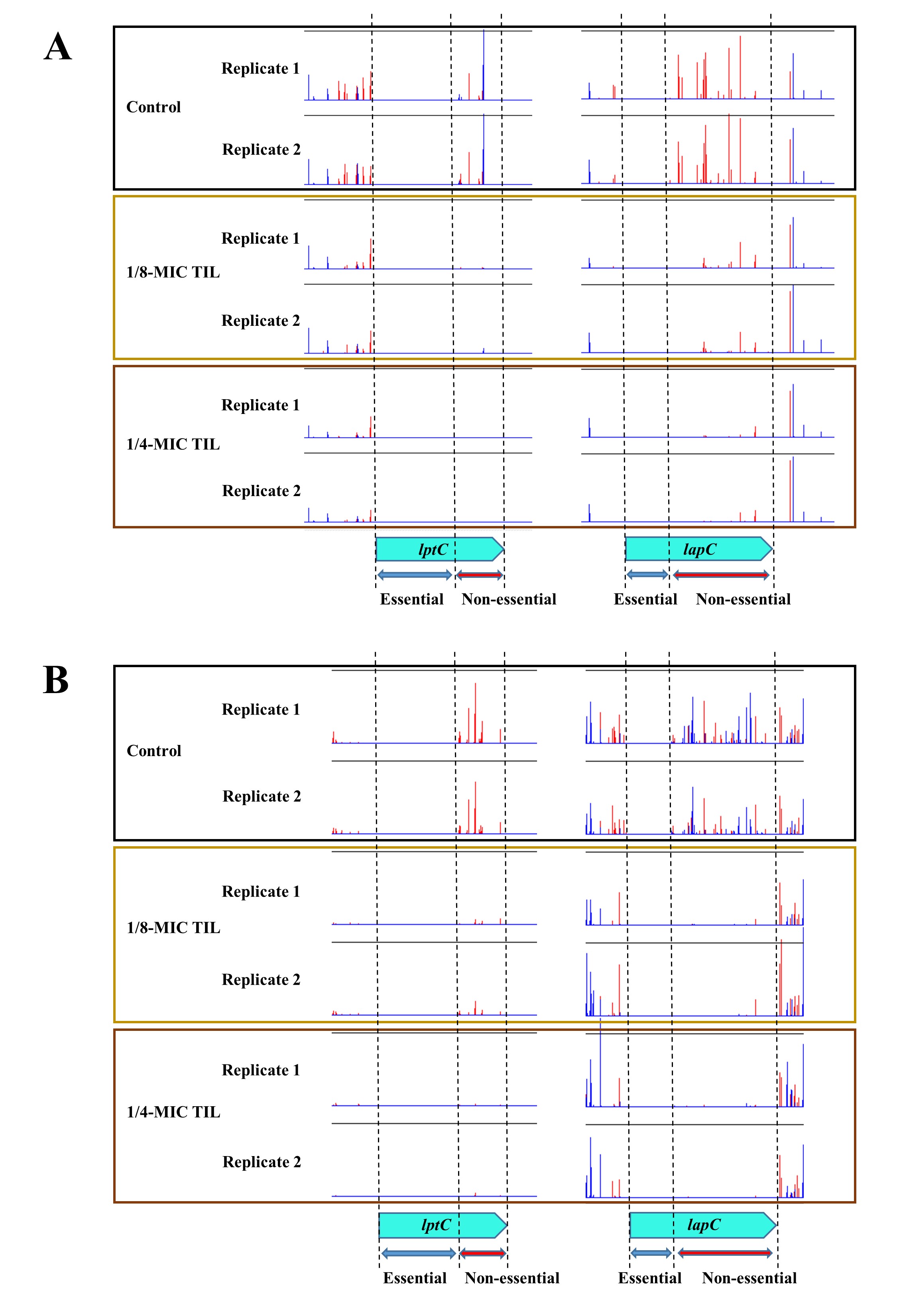


**FIGURE S3** Transposon insertion map of *lptC* and *lapC* for ETEC5621 (A) and MG1655 (B). Insertion site reads were plotted on the gene sequences and inspected using the Artemis genome browser. The figure shows that both *lptC* and *lapC* consist of an essential part at the 5’ end containing no insertion and a non-essential part at the 3’ end where insertions are present but depleted following tilmicosin (TIL) exposure.

**TABLE S1** Parameters of TraDIS datasets for the sequencing libraries

| **Library (condition)** | **Total reads** | **Reads mapped (100%)** | **Total UIS^a^** | **Total seq len/Total UIS^b^** |
| --- | --- | --- | --- | --- |
| ETEC5621_vali | 12,608,598 | 92.9 | 290,909 | 19 |
| MG1655_vali | 14,196,675 | 96.1 | 394,647 | 12 |
|  |  |  |  |  |
| ETEC5621_control_1 | 6,397,611 | 97.7 | 165,095 | 33 |
| ETEC5621_control_2 | 6,586,774 | 97.1 | 170,234 | 32 |
| ETEC5621_1/8MIC_1 | 6,141,181 | 97.3 | 164,611 | 33 |
| ETEC5621_1/8MIC_2 | 6,650,093 | 97.1 | 176,540 | 30 |
| ETEC5621_1/4MIC_1 | 6,727,950 | 95.9 | 181,883 | 30 |
| ETEC5621_1/4MIC_2 | 7,007,532 | 97.6 | 174,366 | 31 |
| MG1655_control_1 | 7,247,888 | 94.9 | 369,063 | 13 |
| MG1655_control_2 | 6,085,995 | 97.9 | 330,129 | 14 |
| MG1655_1/8MIC_1 | 6,562,637 | 96.9 | 342,582 | 14 |
| MG1655_1/8MIC_2 | 9,616,171 | 93.8 | 399,845 | 12 |
| MG1655_1/4MIC_1 | 8,364,600 | 97.7 | 346,464 | 13 |
| MG1655_1/4MIC_2 | 7,501,339 | 96.1 | 340,499 | 14 |

^a^Total UIS, the total number of unique insertion site.

^b^Total seq len/Total UIS, the average number of nucleotides for the occurrence of one unique insertion.

**TABLE S2** MICs (µg/ml) of ampicillin/sulbactam (AMP/SUL), gentamycin (GEN), tetracycline (TET), ciprofloxacin (CIP) and colistin (COL) in the wild-type and gene-deletion mutants of ETEC5621

| **Genotype** | **AMP/SUL^a^** | **GEN** | **TET** | **CIP** | **COL** |
| --- | --- | --- | --- | --- | --- |
| Wild-type | 32 | 0.5 | 0.5 | 0.008 | 0.125 |
| Δ*surA* | 8 | 0.5 | 0.25 | 0.001 | 0.03 |
| Δ*waaG* | 32 | 1 | 0.5 | 0.008 | 0.06 |
| Δ*waaP* | 32 | 0.5 | 0.5 | 0.008 | 0.06 |
| Δ*bamB* | 16 | 1 | 0.25 | 0.004 | 0.125 |
| Δ*pgm* | 16 | 2 | 0.5 | 0.008 | 0.125 |
| Δ*acrA* | 32 | 0.5 | 0.125 | 0.002 | 0.06 |
| Δ*acrB* | 32 | 1 | 0.125 | 0.002 | 0.125 |
| Δ*tolC* | 32 | 1 | 0.125 | 0.002 | 0.125 |
| Δ*prc* | 16 | 0.5 | 0.25 | 0.004 | 0.06 |
| Δ*nlpI* | 16 | 0.5 | 0.5 | 0.002 | 0.125 |

^a^AMP/SUL, Ampicillin/sulbactam in a 2:1 ratio tested using the Sensititre™ Gram Negative MIC Plate (ThermoFisher Scientific, USA).

**TABLE S3** List of primers used in the study

| **Name** | **Description** | **Sequence** **(5'-3')** |
| --- | --- | --- |
| SurA_KO_F | Forward primer for *surA* deletion | TGTTGATTTACCACGTAATCCGCAGTGCGGTTAATTGAAATGGAAAAAGTGTGTAGGCTGGAGCTGCTTC |
| SurA_KO_R | Reverse primer for *surA* deletion | AATCCCGGCGGGCTCGCCGGGAGTGATCACAACACGTTGGGTTTTAACCAATGGGAATTAGCCATGGTCC |
| lptC_KO_F | Forward primer for *lptC* deletion | TTATTACTCCTGGCGCAGGGCAAACTGGATGAAGCCAAAGGGCAATCGATGTGTAGGCTGGAGCTGCTTC |
| lptC_KO_R | Reverse primer for *lptC* deletion | CGGCAAATGCCGGAATGCTGGCGGCCAGAAGTGAGCTGGCAAGCACAAGAATGGGAATTAGCCATGGTCC |
| acrA_KO_F | Forward primer for *acrA* deletion | TTAACTTTTGACCATTGACCAATTTGAAATCGGACACTCGAGGTTTACATGTGTAGGCTGGAGCTGCTTC |
| acrA_KO_R | Reverse primer for *acrA* deletion | TCGGGCGATCGATAAAGAAATTAGGCATGTCTTAACGGCTCCTGTTTAAGATGGGAATTAGCCATGGTCC |
| acrB_KO_F | Forward primer for *acrB* deletion | TGCTCAGCCTGAACAGTCCAAGTCTTAACTTAAACAGGAGCCGTTAAGACGTGTAGGCTGGAGCTGCTTC |
| acrB_KO_R | Reverse primer for *acrB* deletion | GTTATGCATAAAAAAGGCCGCTTACGCGGCCTTAGTGATTACACGTTGTAATGGGAATTAGCCATGGTCC |
| tolC_KO_F | Forward primer for *tolC* deletion | AATTTTACAGTTTGATCGCGCTAAATACTGCTTCACCACAAGGAATGCAAGTGTAGGCTGGAGCTGCTTC |
| tolC_KO_R | Reverse primer for *tolC* deletion | ATCTTTACGTTGCCTTACGTTCAGACGGGGCCGAAGCCCCGTCGTCGTCAATGGGAATTAGCCATGGTCC |
| bamB_KO_F | Forward primer for *bamB* deletion | CGAAATGATGCAGATGAAAATTAATAATTTGTCCATCTGAGAGGGACCCGGTGTAGGCTGGAGCTGCTTC |
| bamB_KO_R | Reverse primer for *bamB* deletion | GAAAACGGCCCCTGTCCAGGAGCCGTTTTCAAAGTGAACGACAGAGACGAATGGGAATTAGCCATGGTCC |
| lapC_KO_F | Forward primer for *lapC* deletion | GTCCTCTATCAACGAAGACAAAGCGCACTAAGGGAAACAGATAACAGGTTGTGTAGGCTGGAGCTGCTTC |
| lapC_KO_R | Reverse primer for *lptc* deletion | TTTCCACACCGATTGCAAGTAAGATATTTCGCTAACTGATTTATAATTAAATGGGAATTAGCCATGGTCC |
| pgm_KO_F | Forward primer for *pgm* deletion | TGAGAAGGTTTGCGGAACTATCTAAAACGTTGCAGACAAAGGACAAAGCAGTGTAGGCTGGAGCTGCTTC |
| pgm_KO_R | Reverse primer for *pgm* deletion | ATACGTAAAAAAGGGCGATCTTGCGACCGCCCTTTTTTTATTAAATGTGTATGGGAATTAGCCATGGTCC |
| waaP_5621_KO_F | Forward primer for *waaP* deletion in ETEC5621 | TTTATACAGTCTGCCAGAGAAAGCGGCGGATATCATAACGGGTGGTCTGGGTGTAGGCTGGAGCTGCTTC |
| waaP_5621_KO_R | Reverse primer for *waaP* deletion in ETEC5621 | ATCATCTCTTGTGGATTAAAATAGTGGGCACTCATATTTCTCTCCGGAAAATGGGAATTAGCCATGGTCC |
| waaP_MG1655_KO_F | Forward primer for *waaP* deletion in MG1655 | TTTGTATAGCTTGCCAGAAAAAGCCGCGGATATCATTACAGGTGGTTTAGGTGTAGGCTGGAGCTGCTTC |
| waaP_MG1655_KO_R | Reverse primer for *waaP* deletion in MG1655 | AAATAAATAGTCATAATAAAGTTAGTTCCAGTACATACTAATAAATATTTATGGGAATTAGCCATGGTCC |
| waaG_5621_KO_F | Forward primer for *waaG* deletion in ETEC5621 | CTGCTGTCGATAAATTACTGCCCTCCTCCACGACAGGTACGTCGTTATGAGTGTAGGCTGGAGCTGCTTC |
| waaG_5621_KO_R | Reverse primer for *waaG* deletion in ETEC5621 | GGATCTTTACCGCGCCATAACGTGGCAAACGGCTCTTTAAGTTCAACCATATGGGAATTAGCCATGGTCC |
| waaG_MG1655_KO_F | Forward primer for *waaG* deletion in MG1655 | AGCTGTTGCCAGAAGATGCCCCTTCAGCTGACAGGAATGCACAATTATGAGTGTAGGCTGGAGCTGCTTC |
| waaG_MG1655_KO_R | Reverse primer for *waaG* deletion in MG1655 | GCATCTTTACCACGCCAAAGTGTGGCAAGCGGCTCTTTTAATTCAACCATATGGGAATTAGCCATGGTCC |
| prc_KO_F | Forward primer for *prc* deletion | GCAGAACACCTGGTGTTCTGAAACGGAGGCCGGGCCAGGCTGTAGGCTGGAGCTGCTTC |
| prc_KO_R | Reverse primer for *prc* deletion | TGTTAAAAAATCAGGCACAATTTCTTGTGCCTGATTGATACATATGAATATCCTCCTTAG |
| nlpI_KO_F | Forward primer for *nlpI* deletion | AACAGGACGTTCATTCAACCGTGGTCTTCGGGAGTGGGAATGTAGGCTGGAGCTGCTTC |
| nlpI_KO_R | Reverse primer for *nlpI* deletion | ATGGCAATCAAAAAAGATTACGGGCTGATGTGTACGTCAGCATATGAATATCCTCCTTAG |
| SurA_veri_F | Forward primer for Δ*surA* verification | TGTTGATTTACCACGTAATCCGCAG |
| SurA_veri_R | Reverse primer for Δ*surA* verification | AATCCCGGCGGGCTCGCCGGGAGTG |
| lptC_veri_F | Forward primer for Δ*lptC* verification | TTATTACTCCTGGCGCAGGGCAAAC |
| lptC_veri_R | Reverse primer for Δ*lptC* verification | CGGCAAATGCCGGAATGCTGGCGGC |
| acrA_veri_F | Forward primer for Δ*acrA* verification | TTAACTTTTGACCATTGACCAATTT |
| acrA_veri_R | Reverse primer for Δ*acrA* verification | TCGGGCGATCGATAAAGAAATTAGG |
| acrB_veri_F | Forward primer for Δ*acrB* verification | TGCTCAGCCTGAACAGTCCAAGTCT |
| acrB_veri_R | Reverse primer for Δ*acrB* verification | GTTATGCATAAAAAAGGCCGCTTAC |
| tolC_veri_F | Forward primer for Δ*tolC* verification | AATTTTACAGTTTGATCGCGCTAAA |
| tolC_veri_R | Reverse primer for Δ*tolC* verification | ATCTTTACGTTGCCTTACGTTCAGA |
| bamB_veri_F | Forward primer for Δ*bamB* verification | CGAAATGATGCAGATGAAAATTAAT |
| bamB_veri_R | Reverse primer for Δ*bamB* verification | GAAAACGGCCCCTGTCCAGGAGCCG |
| lapC_veri_F | Forward primer for Δ*lapC* verification | GTCCTCTATCAACGAAGACAAAGCG |
| lapC_veri_R | Reverse primer for Δ*lapC* verification | TTTCCACACCGATTGCAAGTAAGAT |
| pgm_veri_F | Forward primer for Δ*pgm* verification | TGAGAAGGTTTGCGGAACTATCTAA |
| pgm_veri_R | Reverse primer for Δ*pgm* verification | ATACGTAAAAAAGGGCGATCTTGCG |
| prc_veri_F | Forward primer for Δ*prc* verification | ACATTTCAGCTCTGACTGTC |
| prc_veri_R | Reverse primer for Δ*prc* verification | TTCTTTTTACCTCAGTTTAA |
| nlpI_veri_F | Forward primer for Δ*nlpI* verification | AATCGCTGACAAACGCGTTG |
| nlpI_veri_R | Reverse primer for Δ*nlpI* verification | TGCCTCAAAATTAATGGCGG |
| waaP_5621_veri_F | Forward primer for Δ*waaP* verification in ETEC5621 | TTTATACAGTCTGCCAGAGAAAGCG |
| waaP_5621_veri_R | Reverse primer for Δ*waaP* verification in ETEC5621 | ATCATCTCTTGTGGATTAAAATAGT |
| waaP_MG1655_veri_F | Forward primer for Δ*waaP* verification in MG1655 | TTTGTATAGCTTGCCAGAAAAAGCC |
| waaP_MG1655_veri_R | Reverse primer for Δ*waaP* verification in MG1655 | AAATAAATAGTCATAATAAAGTTAG |
| waaG_5621_veri_F | Forward primer for Δ*waaG* verification in ETEC5621 | ATTGCTGCTGTCGATAAATTACTGC |
| waaG_5621_veri_R | Reverse primer for Δ*waaG* verification in ETEC5621 | CCTCAAAAGGATCTTTACCGCGCCA |
| waaG_MG1655_veri_F | Forward primer for Δ*waaG* verification in MG1655 | GAAAAGCTGTTGCCAGAAGATGCCC |
| waaG_MG1655_veri_R | Reverse primer for Δ*waaG* verification in MG1655 | CTGCAAAAGCATCTTTACCACGCCA |
| Cat_veri_R | Reverse primer for Chl^R^ cassette verification | CACTGGATATACCACCGTTGATATATC |
| pACYC184_F | Forward primer for amplification of pACYC184 backbone | TTTTTTTAAGGCAGTTATTGGTGCC |
| pACYC184_R | Reverse primer for amplification of pACYC184 backbone | TTTAGCTTCCTTAGCTCCTGAAAAT |
| pACYC184_veri_F | Forward primer for pACYC184 verification | CACTTCCCTGTTAAGTATCT |
| surA_C_F | Forward primer for amplification of *surA* complementation | CAGGAGCTAAGGAAGCTAAAATGAAGAACTGGAAAACGCT |
| surA_C_R | Reverse primer for amplification of *surA* complementation | CAATAACTGCCTTAAAAAAATTAGTTGCTCAGGATTTTAA |
| surA_C_veri_R | Reverse primer for verification of *surA* complementation | CATGATCAAACGTTCCATGA |
| 5621waaG_C_F | Forward primer for amplification of *waaG* complementation in ETEC5621 | CAGGAGCTAAGGAAGCTAAAATGATCGTTGCTTTTTGTTT |
| 5621waaG_C_R | Reverse primer for amplification of *waaG* complementation in ETEC5621 | CAATAACTGCCTTAAAAAAATCAACCATCCAGACCACCCG |
| 5621waaG_C_veri_R | Reverse primer for verification of *waaG* complementation in ETEC5621 | CATAATAAACGTCCAGCCCC |
